# Acquired choledochal cyst following intraabdominal tumor surgical excision: A case report

**DOI:** 10.1016/j.amsu.2020.08.001

**Published:** 2020-08-07

**Authors:** Gunadi, Ramadhita, Aditya Rifqi Fauzi, Devy Melati, Elena Sophia Elekta Dilean Siahaan, Eddy Daryanto, Desy Rusmawatiningtyas, Wahyu Damayanti

**Affiliations:** a Pediatric Surgery Division, Department of Surgery, Faculty of Medicine, Public Health and Nursing, Universitas Gadjah Mada/Dr. Sardjito Hospital, Yogyakarta, 55281, Indonesia; b Pediatric Surgery Division, Department of Surgery, Banyumas General Hospital, Banyumas, 53192, Indonesia; c Department of Child Health, Faculty of Medicine, Public Health and Nursing, Universitas Gadjah Mada/Dr. Sardjito Hospital, Yogyakarta, 55281, Indonesia

**Keywords:** Acquired choledochal cyst, Surgical complication, Intraabdominal tumor excision, Roux-en-Y hepaticojejunostomy

## Abstract

**Introduction:**

Choledochal cyst (CC) is a morphological malformation characterized by dilatations of the biliary tree that might present later with clinical symptoms, including jaundice, abdominal pain or pancreatitis.

**Presentation of case:**

Here, we reported a 10-month-old female infant with CC presenting with jaundice and a right upper quadrant mass and who was malnourished following a surgical excision of retroperitoneal teratoma one month ago. Laboratory findings were total bilirubin of 14.17 mg/dL, direct bilirubin of 12.24 mg/dL, gamma glutamyl transferase of 1157 U/L, and alkaline phosphatase 187 U/L. Abdominal computed tomography scan showed a CC that caused dilatation of the proximal common bile duct (CBD), common hepatic duct, and intrahepatic bile duct. We decided to perform an explorative laparotomy and found a CC with diameter of 5 cm. Then, we conducted a Roux-en-Y hepaticojejunostomy. Due to hepaticojejunostomy anastomosis leakage, relaparotomies were done. The patient was uneventfully discharged 17 days after the third surgery.

**Discussion:**

Our findings are unique because the patient had a normal biliary tree previously and underwent intraabdominal tumor surgery. Notably, besides being an acquired CC, our case might be due to inadvertent bile duct ligation during the first operation or bile duct obstruction as a complication of the first operation.

**Conclusions:**

CC should be considered as a potential complication of intraabdominal tumor excision, especially if its location is near the CBD. Roux-en-Y hepaticojejunostomy is still the best choice for CC management.

## Highlights

• The development of acquired choledcohal cyst (CC) might be due to disruption of the biliary tree especially the common bile duct during the excision of intraabdominal tumor.
• Abdominal computed tomography scan is useful and highly accurate to diagnose of CC.
• Roux-en-Y hepaticojejunostomy is still the best choice for CC management.

## 1 Introduction

Choledochal cyst (CC) is a morphological disorder characterized by dilatations of the biliary tree and clinical features which might present later, including jaundice, pancreatitis, or nonspecific abdominal pain [[1], [2], [3], [4]]. Its incidence is 1 in 13,000 and 1 in 100,000 in Asian and Western populations, respectively [1]. Most CC cases (80%) are reported in infants and children [1], but interestingly, its incidence has increased in adults (approximately 20%) [[5], [6], [7]]. These findings might be related to the advancement of diagnostic imaging [7], however, they are raising the hypothesis that the etiology of CC might not always be congenital, but can also be acquired [8,9]. Here, we reported a case of CC in a child following a surgical excision of an intraabdominal tumor, along with its possible cause and management. This case report has been reported in line with the SCARE 2018 criteria [10].

## 2 Presentation of Case

**Fig. 1:**
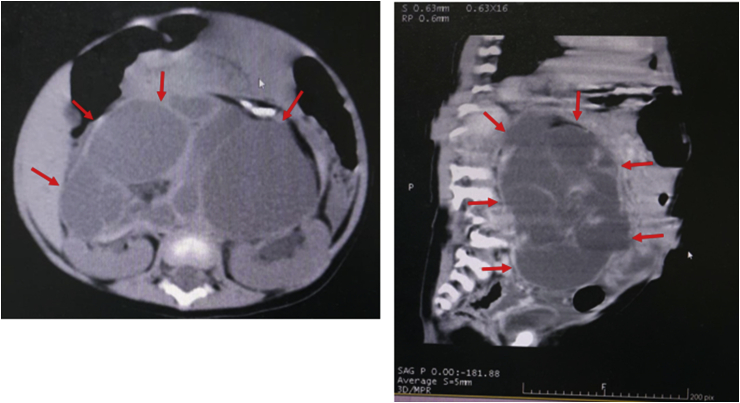
Abdominal CT scan revealed a complex cystic mass measuring approximately 11.4 × 10.9 × 7.3 cm and normal common bile duct (CBD).

A 10-month-old female infant was referred to our hospital due to jaundice and right upper quadrant mass. A month before being admitted to our hospital, she underwent surgery in another hospital due to an intraabdominal tumor. Her abdominal computed tomography (CT) scan showed a complex cystic mass located in front of the kidneys and measuring approximately 11.4 × 10.9 × 7.3 cm, and normal common bile duct (CBD) (Fig. 1). She had undergone an explorative laparotomy for tumor excision. The tumor was located in the retroperitoneal space and completely resected. The CBD was confirmed normal at the time of the tumor excision. Eventually, the histopathological findings of the tumor showed mature teratoma.

**Fig. 2:**
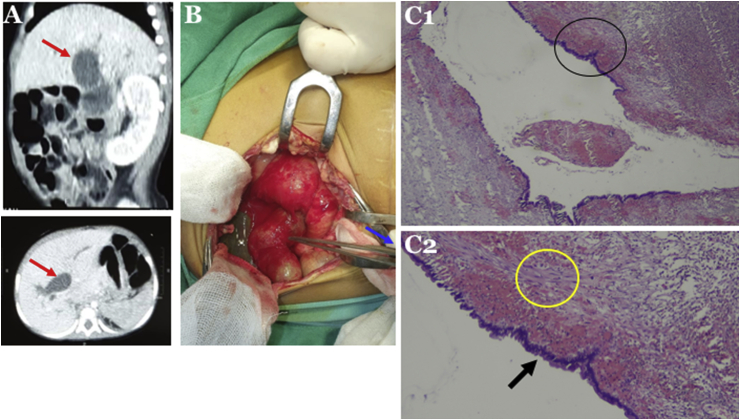
(a) Abdominal CT scan showed a choledochal cyst (CC) that caused dilatation of the proximal CBD, common hepatic duct, and intrahepatic bile duct (arrow); (b) CC with diameter of 5 cm was found during explorative laparotomy (arrow); (c) Histopathological findings showed walls that consisted of thick fibrous tissue (yellow circle) and focal columnar epithelium (arrow), confirmed as CC. 1, original magnification x100; 2, x400. (For interpretation of the references to colour in this figure legend, the reader is referred to the Web version of this article.)

One week after the surgical excision, the patient became jaundiced, had pale stool, dark urine and had become malnourished. Due to no improvement after conservative management in the previous hospital, she was referred to our hospital. Her laboratory findings were total bilirubin of 14.17 mg/dL, direct bilirubin of 12.24 mg/dL, gamma glutamyl transferase of 1157 U/L, and alkaline phosphatase 187 U/L. Abdominal CT scan showed a CC that caused dilatation of the proximal CBD, common hepatic duct, and intrahepatic bile duct (Fig. 2a).

We decided to perform an explorative laparotomy and found a CC with diameter of 5 cm (Fig. 2b). We, then, conducted a Roux-en-Y hepaticojejunostomy. Histopathological findings showed walls that consisted of thick fibrous tissue and focal columnar epithelium, confirmed as CC (Fig. 2c).

At 8 days after surgery, the patient suffered fever and abdominal tenderness, and was diagnosed with general peritonitis due to suspected anastomosis leakage. We decided to perform early procedures for fixing the suspected leakage since the patients’ condition was getting worse and she had a feeding intolerance. During the explorative laparotomy, a hepaticojejunostomy leakage was confirmed and re-anastomosis was performed, while the jejunojejunostomy anastomosis was intact. Unfortunately, at 11 days following the second surgery, the patient had a fever and abdominal tenderness with feeding intolerance. We decided to conduct the third laparotomy due to general peritonitis. We found fecal material within the abdominal cavity and hepaticojejunostomy anastomosis leakage. Subsequently, we conducted re-anastomosis. We did not use prosthetic materials, such as probes, during the re-laparotomies. Finally, she was uneventfully discharged from our hospital 17 days after the last surgery.

The last follow-up of the patient in our outpatient clinic was at two months after surgery, and she showed neither icterus nor abdominal complaints, and was doing well. Due to no symptoms nor signs of icterus and abdominal complaints, we did not perform any additional examinations on the patient during the last follow-up. Therefore, we were unable to evaluate the intrahepatic bile ducts anatomy after the CC excision.

## 3 Discussion

We reported a CC case in a child after surgical excision of intraabdominal tumor. To the best of our knowledge, this case is the first report of CC after intraabdominal tumor excision. The development of an acquired CC in our patient might be due to disruption of the biliary tree especially the CBD during the tumor excision. So far, there are no studies about the possible association or any associated syndrome between the first diagnosis of tumor (*i.e.* teratoma) and the following diagnosis of CC. Interestingly, one previous study reported two cases of mature cystic teratoma at the porta hepatis that were incorrectly pre-operatively diagnosed as CC [11]. Moreover, three patients with retroperitoneal teratoma who were accompanied by Down syndrome (n = 2) and Klinefelter syndrome (n = 1) have been reported [12].

There are several hypotheses for the etiology of an acquired CC as follows: 1) acquired dilatations resulting from complications of anomalous pancreaticobiliary ductal union; 2) history of previous surgery; and 3) viral infection [8,9]. Moreover, two-thirds of CC are either acquired or a combination of congenital and acquired [13]. Our findings are unique because the patient had a normal biliary tree previously and underwent intraabdominal tumor surgery. Notably, besides being an acquired CC, our case might be due to inadvertent bile duct ligation during the first operation or bile duct obstruction as a complication of the first operation. It has been reported that removal of the retroperitoneal teratoma might be risky due to several reasons, including the tumor extent, the surrounding structures displacement, and the tumor adhesion to adjacent tissues, resulting in perioperative complications, such as choledochal tear [12].

Although the second abdominal CT scan was able to show a CC in our case, the magnetic resonance cholangiopancreatography should be performed instead to give more information about the biliary dilatation, the anatomy of the pancreatic duct, the underlined cause of the jaundice, the CC type, and any associated anomalies [1,7], which would have helped in the operative strategy so the laparotomy was not exploratory but targeted.

CC is classified into 4 types: 1) type I, a fusiform/spherical dilatation of the extrahepatic bile duct system; 2) type II, a diverticular dilatation of the extrahepatic biliary tree; 3) type III, a cyst located within the duodenal wall; 4) type IV, multiple cysts within the intra- and the extrahepatic biliary system; and 5) type V (Caroli disease), dilatations of the intrahepatic bile duct [1]. More than 95% of CC are types I and IV [14].

Although she twice underwent re-laparotomies due to anastomosis leakage, our patient was finally discharged uneventfully from the hospital after undergoing Roux-en-Y hepaticojejunostomy. Cyst excision and restoring biliary enteric drainage is the preferred management for CC over internal drainage surgeries because of low morbidity and lower risk of malignant degeneration [1]. The two most common methods performed are hepaticoduodenostomy and Roux-en-Y hepaticojejunostomy (RYHJ) [1]. Due to the significantly higher incidence of bile reflux in hepaticoduodenostomy, RYHJ is now considered preferable than hepaticoduodenostomy [1,[15], [16], [17]]. The surgical procedure objectives for CC include: a) the cyst wall and the gall bladder excision to decrease the malignancy risk; b) pancreatic-biliary reflux prevention; and c) the biliary-enteric channel reconstruction for optimal bile drainage [5,15]. Most CC patients (>85–90%) show good outcomes following Roux-en-Y surgery, whereas only 6–10% patients have complications, including anastomosis leakage [16,18,19].

The failure of hepaticojejunostomy anastomosis in our patient might be due to her malnourished condition. There are several factors that can affect the anastomosis healing process, consisting of the primary disease, presence of infection, and patient's nutritional status [20].

## 4 Conclusions

CC should be considered as a potential complication of intraabdominal tumor excision, especially if its location is near the common bile duct. Roux-en-Y hepaticojejunostomy is still the best choice for CC management.

## Ethical approval

The informed consent form was declared that patient data or samples will be used for educational or research purposes. Our institutional review board also do not provide an ethical approval in the form of case report.

## Sources of funding

The article processing charge of this article was funded by the Faculty of Medicine, Public Health and Nursing, Universitas Gadjah Mada.

## Author contribution

Gunadi conceived the study. Gunadi and Aditya Rifqi Fauzi drafted the manuscript. Ramadhita, Devy Melati, Elena Sophia Elekta Dilean Siahaan, Eddy Daryanto, Desy Rusmawatiningtyas, and Wahyu Damayanti critically revised the manuscript for important intellectual content. Gunadi, Ramadhita, Aditya Rifqi Fauzi, Devy Melati, Elena Sophia Elekta Dilean Siahaan, Eddy Daryanto, Desy Rusmawatiningtyas, and Wahyu Damayanti facilitated all project-related tasks.

## Research registration number

The manuscript is a case report, not considered a formal research involving participants.

## Guarantor

Gunadi.

## Consent

Written informed consent was obtained from the parent of the patient for publication of this case report and accompanying images. A copy of the written consent is available for review by the Editor-in-Chief of this journal on request.

## Provenance and peer review

Not commissioned, externally peer reviewed.

## Declaration of competing interest

No potential conflicts of interest were declared.
